# Identification, Transcriptome, and Proteome Analysis of Expansin-like Subfamilies in the Storage Root Across *I. trifida* (2x), Wild (4x, 6x) and Cultivated Sweet Potatoes

**DOI:** 10.3390/plants15020305

**Published:** 2026-01-20

**Authors:** Jingjing Li, Zhiyu Zhang, Qiuzhuo Li, Chunli Geng, Haoxi Huang, Xiaojian Qin, Yongshu Liang, Wenbin Nan, Hanma Zhang, Yufan Fu, Ming Li

**Affiliations:** 1 Chongqing Key Laboratory of Plant Environmental Adaptation Biology, College of Life Sciences, Chongqing Normal University, Chongqing 401331, China; 15012064421@163.com (J.L.); m15911789910@163.com (Z.Z.); 15924846627@163.com (C.G.); 18983350449@163.com (H.H.); qinxiaojian@cqnu.edu.cn (X.Q.); yongshu-liang@yeah.net (Y.L.); nanwenbin513@163.com (W.N.); hanmazhang@126.com (H.Z.); 2 Chongqing Three Gorges Academy of Agricultural Sciences, Wanzhou 404155, China; lqz380@163.com; 3 Engineering and Technology Research Center for Sweetpotato of Chongqing, School of Life Science, Southwest University, Chongqing 400715, China; xsfanyufu@126.com

**Keywords:** sweet potato, wild relatives, expansin-like subfamilies, identification, transcriptome, proteome, storage root development

## Abstract

The expansin-like subfamilies (EXLA and EXLB) are vital for plant cell wall dynamics, but it remains uncharacterized in wild tetraploid and hexaploid *Ipomoea batatas*, and its role in the storage root (SR) development is poorly understood. In this work, we identified 4, 3, 3, and 3 EXLAs, alongside 11, 9, 13, and 8 EXLBs, in diploid *I. trifida* strain Y22, wild tetraploid *I. batatas* strain Y428B, and hexaploid *I. batatas* strain Y601, and cultivated sweet potato ‘Nancy Hall’, respectively. A comprehensive bioinformatic analysis of the expansin-like genes and proteins was performed to reveal their potential roles in SR development. Gene expression profiling showed that *EXLA* members were expressed during SR development, while approximately half of the *EXLB* members were expressed in Y22, Y428B (pencil root), Y601, and NH, respectively. Proteomic analysis (4D-DIA) detected 2, 1, 1, and 1 EXLAs, and 3, 3, 3, and 3 EXLBs in the mature SRs of the respective species. Integrated transcriptomic and proteomic analyses suggested that downregulating *Iba6xEXLB2* and *Iba6xEXLB1* may be associated with SR swelling in sweet potato. Furthermore, subcellular localization assays confirmed that Iba6xEXLB2 and Iba6xEXLB8 are localized to the cell wall/membrane. This study enhances the understanding of the expansin-like gene subfamily in sweet potato and its wild relatives and lays the groundwork for future functional studies on the role of expansin-like genes in SR development.

## 1. Introduction

The expansin protein is an important cell wall relaxation factor with non-enzymatic activity. Under acidic conditions, it disrupts hydrogen bonds between hemicellulose and cellulose microfibrils, after which the cellulose is loosened by cell turgor pressure [1]. Cells synthesize new wall substances to fill into the loosening structure, thereby achieving wall expansion and cell growth [2]. It is widely present in higher plants, bacteria, and fungi. Typical plant expansin proteins are torpedo-shaped and contain DPBB_1 and Pollen_allerg_1 domains preceded by a signal peptide [3]. Through phylogenetic analysis, expansin proteins can be classified into four subfamilies, namely α-expansin (EXPA), β-expansin (EXPB), expansin-like A (EXLA), and expansin-like B (EXLB) [3].

Expansin was first discovered and named in cucumber [1]. To date, expansin proteins have been identified in many crops, such as 58, 241, 88, 75, and 93 expansin members that have been identified in rice [3], maize [4], common wheat [5], soybean [6], and cotton [7], respectively. In addition to participating in cell growth, expansin is also involved in the adaptive response of organisms to environmental stimuli, as well as in various physiological processes related to the growth and development of the cell wall, such as plant morphogenesis, biotic and abiotic stress responses, fruit ripening and softening, and pollen tube elongation [2]. For instance, rice *OsEXPA10* and *Petunia hybrida PhEXPA1* regulate its root cell elongation and cell size, respectively [8,9]. The wheat *TaEXPA2* and *TaEXPA7-B* [10,11], soybean *GsEXPB1* and *GsEXLB14* [12,13], poplar *PtEXLA1* [14], and sweet osmanthus *OfEXLA1* [15], can regulate plant resistance to drought or salt stress, respectively. Overexpression of the apple *MdEXLB1* reduced plant height, and accelerated the ripening and texture softening process of transgenic tomato [16]. One developmental and the other environmental or hormonal signaling pathways, they converge to modulate the expression of *A. thaliana AtEXP7* and *AtEXP18* [17].

Sweet potato (*Ipomoea batatas*) is a globally significant food crop, its economic yield primarily deriving from its storage roots (SRs), the swelling efficiency of which directly determines the final yield. The formation and swelling of sweet potato SRs constitute a complex process that progresses sequentially from primary to secondary and then to tertiary growth. During the tertiary growth phase, the accessory cambium, unique to sweet potato, generates a large number of parenchyma cells [18,19]. The rapid root thickening is achieved through the continuous proliferation and volume expansion of these cells. Cell wall loosening serves as the physical prerequisite for cell volume expansion; therefore, the dynamic regulation of cell wall structure plays a central role in storage root development. Expansin, as a non-enzymatic wall-loosening protein [1], is involved in the regulation of sweet potato storage root swelling. In a previous study, Li et al. and Zhang et al. reported 37 and 59 expansin proteins in *Ipomoea trifida* 2x and sweet potato, respectively [20,21]. Two expansin genes, namely *IbEXP1* [22] and *IbEXPA4* [23], have been reported that they have the same function and both can promote the length of FRSS (fibrous root between SR and underground stem) in sweet potato. Downregulation of the *IbEXP1* gene shortened the FRSS and enhanced SR development [22]. While these reports establish the importance of expansins in SR development, overall knowledge is limited, and this is especially true for the specific functions of the EXLA and EXLB subfamilies, which remain largely unexplored in sweet potato.

Among the 600–700 wild relative species, *Ipomoea leucantha*, *Ipomoea tenuissima*, *I. trifida*, *Ipomoea littoralis*, *Ipomoea tabascana*, and *Ipomoea triloba* were once considered as potential progenitors of sweet potato [24]. *I. trifida* 2x is the closest and most likely diploid progenitor of sweet potato [25,26]. Kyndt et al. were the first to discover the natural transgenic elements *Ib*T-DNA1 and *Ib*T-DNA2 in sweet potato; additionally, *Ib*T-DNA2 was detected in *Ipomoea trifida* 2x and *I. batatas* 4x [27]. Subsequently, *Ib*T-DNA1 was found in *Ipomoea cordatotriloba* and *Ipomoea tenuissima*, and both *Ib*T-DNA1 and *Ib*T-DNA2 were found in *I. batatas* 4x [28]. Combining whole-genome variations, *Ib*T-DNAs sequences, homologous haplotype sequences, and chloroplast genomes, Yan et al. revealed that the diploid progenitor and tetraploid progenitor of sweet potato are *Ipomoea aequatoriensis* and *I. batatas* 4x, which donated the B1 and B2 subgenome, respectively; and that sweet potato originated from the crossing between the diploid and tetraploid progenitors, followed by the whole-genome duplication from the triploid [29]. Furthermore, Colombia hybrid 4x, such as K500-1, may have evolved from *I. trifida* 2x [29]. Based on previous studies, the evolutionary history of sweet potato is complex. There might also have been a wild hexaploid form of sweet potato somewhere, which then evolved into cultivated types. Obviously, *I. trifida* 2x and wild *I. batatas* are key wild relatives in the evolution of sweet potato. However, research on expansins, particularly the EXLA and EXLB subfamilies, in wild relatives of sweet potato, remains very limited.

In this work, we selected the typical SR-forming materials, including *I. trifida* 2x strain Y22 (accession: CIP 698014) [19], wild *I. batatas* 6x strain Y601 [29], and the cultivated sweet potato variety Nancy Hall (NH), and the pencil root material wild *I. batatas* (4x) multiple hybrid strain Y428B (accession: CIP 695150B) [29], for bioinformatic, gene expression, and protein expression analyses of the EXLA and EXLB. Through systematic analysis, we aim to reveal the gene and protein expression patterns of expansin-like subfamilies during SR development, thereby provide a theoretical basis and practical guidance for genetic improvement and promote the utilization of these genes in sweet potato breeding and yield enhancement.

## 2. Results

### 2.1. Identification and Characterization of Expansin-like Family Members

To avoid confusion, hereafter in the current text, *I. trifida* 2x will be referred to as Itr2x, wild *I. batatas* 4x as Iba4x, wild *I. batatas* 6x as Ibw6x, and cultivated *I. batatas* 6x as Iba6x.

Based on 36 *A. thaliana* expansin proteins from TAIR (https://www.arabidopsis.org/), 4, 3, 3, and 3 EXLAs were identified in Y22, Y428B, Y601, and NH, respectively. The EXLAs of these materials contained 258–268 amino acids, and their molecular weights ranged from 27.46 to 29.41 kD. The theoretical pI (isoelectric point) of these EXLAs was between 4.62 and 6.96. The instability index of Itr2xEXLA3, Iba4xEXLA3, Ibw6xEXLA3, and Iba6xEXLA3 was higher than 40.0, indicating that these EXLA3 proteins of the four species were unstable. The aliphatic index and grand average of hydropathicity (GRAVY) ranged from 70.70 to 92.26, and between −0.164 and 0.073, respectively (Table S1).

A total of 11, 9, 13, and 8 EXLBs were identified in Y22, Y428B, Y601, and NH, respectively (Table S1). The number of amino acids ranged from 186 to 588, and molecular weights ranged from 20.69 to 65.79 kD, and the variation range was greater than that of the EXLA proteins. The theoretical pI of these EXLBs ranged from 5.48 to 9.17. Approximately 65.9% of EXLBs belong to alkaline proteins, while all EXLAs are acidic. Most of the EXLBs were unstable, including all EXLBs in cultivated sweet potato NH, but Itr2xEXLB1, Iba4xEXLB3, and Ibw6xEXLB1 from the wild relatives were stable. The aliphatic index of EXLBs ranged from 68.95 to 92.26, the variation range was similar to that of EXLAs. Since the GRAVY values of about 95% of EXLBs in four species (except for Itr2xEXLB7 and Iba4xEXLB6) were negative, this indicates that most EXLBs are hydrophilic proteins (Table S1). Interestingly, Itr2xEXLA2, Iba4xEXLA2, Ibw6xEXLA2, and Iba6xEXLA2 have the same number of amino acids, molecular weight, theoretical pI, instability index, aliphatic index, and GRAVY values.

### 2.2. Gene Localization and Duplication Pattern Analysis

**Figure 1:**
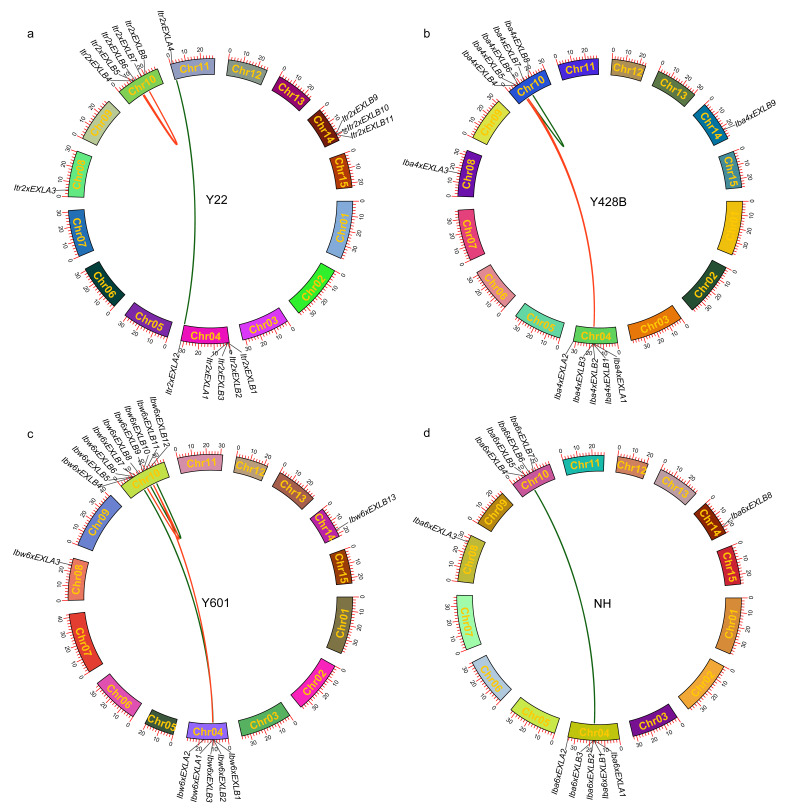
Chromosomal distribution and genome internal collinearity of expansin-like genes. (**a**–**d**) The outer ring of the chromosomes shows the genomic locations of *EXLAs* and *EXLBs*, and the lines between chromosomes represent the syntenic relationships of expansin-like genes in the Y22, Y428B, Y601, and NH genomes, respectively. The red lines indicate syntenic gene pairs with two-to-one or one-to-two relationships, while the green lines indicate one-to-one syntenic gene pairs.

Chromosome localization analysis of *EXLAs* and *EXLBs*, based on GFF3 annotation files, showed that expansin-like genes are distributed mainly on chromosome 4–10 in the four species, but their distribution patterns varied considerably (Figure 1). Among them, *Itr2xEXLAs* in Y22 are located on chromosomes 4, 8, and 11 (Figure 1a). *Iba4xEXLAs* in Y428B, *Ibw6xEXLAs* in Y601, and *Iba6xEXLAs* in NH, are located on chromosomes 4 and 8 of their respective genomes (Figure 1b–d). *Itr2xEXLBs* are located on chromosomes 4, 10, and 14. Most *EXLBs* in Y428B, Y601, and NH are distributed on chromosomes 4 and 10 of their respective genomes, with only one gene located on chromosome 14 (Figure 1b–d). Among these four species, nine *Ibw6xEXLBs* are located on chromosome 10 in Y601 genome, representing the largest number of *EXLB* genes on a single chromosome (Figure 1c). The uneven distribution of expansin-like genes may be due to uneven replication of chromosome fragments.

In the course of genome evolution, diploid *I. trifida* underwent a whole-genome triplication (WGT) event, and cultivated sweet potato underwent twice whole-genome duplications (WGD) [19,30]. Therefore, gene duplication during the WGT/WGD event is a vital driving force for the evolution of gene families. To further reveal the duplication type of expansin-like genes in the four species studied in this work, intra-genome synteny analysis was conducted by MCScanX in TBtools-II [31,32]. The results showed that five tandem duplication gene pairs were found in four genomes, comprising *Itr2xEXLB2*/*Itr2xEXLB3* on chromosome 4 and *Itr2xEXLB5*/*Itr2xEXLB6* on chromosome 10 in the Y22 genome; *Iba4xEXLB2*/*Iba4xEXLB3* on chromosome 4 in the Y428B genome; *Ibw6xEXLB2*/*Ibw6xEXLB3* on chromosome 4 in the Y601 genome; and *Iba6xEXLB2*/*Iba6xEXLB3* on chromosome 4 in the NH genome (Figure 1a–d, Table S2). Eleven WGD or segmental duplication gene pairs were identified in four genomes, including *Itr2xEXLA2*/*Itr2xEXLA4* and the pairs involving *Itr2xEXLB4/Itr2xEXLB7* and *Itr2xEXLB6/Itr2xEXLB7* in the Y22 genome; *Iba4xEXLB1/Iba4xEXLB4*, *Iba4xEXLB1/Iba4xEXLB6*, and *Iba4xEXLB6/Iba4xEXLB7* in the Y428B genome; the pairs involving *Ibw6xEXLA1/Ibw6xEXLB11*, *Ibw6xEXLB8/Ibw6xEXLB11*, *Ibw6xEXLB9/Ibw6xEXLB12*, and *Ibw6xEXLB1/Ibw6xEXLB7* in the Y601 genome; and *Iba6xEXLB1/Iba6xEXLB5* in the NH genome (Figure 1a–d, Table S2). These results indicate that both tandem duplication and WGD or segmental duplication have occurred during the evolution of the expansin-like gene family in the four species (Y22, Y428B, Y601, and NH).

### 2.3. Gene Collinearity Analysis Among Four Species

**Figure 2:**
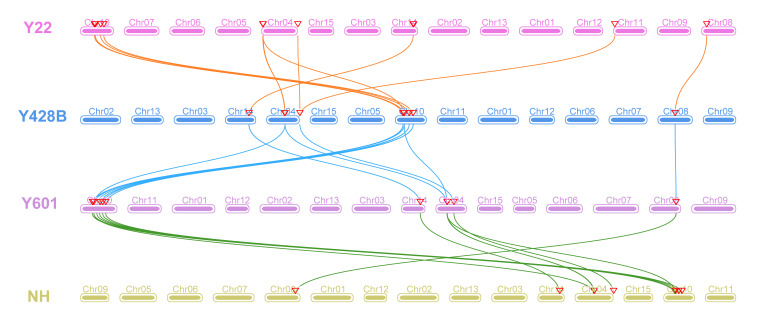
Syntenic relationships of expansin-like genes between four species. Details of the one-to-one collinear gene pairs are listed in Table S3.

To further investigate the evolutionary relationships of *EXLAs* and *EXLBs*, we explored the collinearity relationships between expansin-like genes and orthologous genes in the Y22, Y428, Y601, and NH genomes. A total of 43 expansin genes (79.6%) showed collinear relationships in the Y22-to-Y428B, Y428B-to-Y601, and Y601-to-NH comparisons (Figure 2, Table S3). For example, eight *Iba4xEXLBs* are collinear with ten *Iba6xEXLBs*, and eight *Iba6xEXLBs* are collinear with six *Iba6xEXLBs*. Among them, fourteen gene pairs with two-to-one, one-to-two, or one-to-three collinear relationships were identified between genomes. For instance, *Itr2xEXLA2*/*Itr2xEXLA4* are collinear with *Iba4xEXLA2*, *Iba4xEXLB1* is collinear with *Ibw6xEXLB2*/*Ibw6xEXLB7*, and *Ibw6xEXLB11*/*Ibw6xEXLB4*/*Ibw6xEXLB8* are collinear with *Iba6xEXLB6*. These results indicate that *EXLA* and *EXLB* subfamilies may have expanded and contracted during evolution. Interestingly, we found that all *EXLAs* have a collinearity relationship across the four genomes. For example, *Itr2xEXLA4* is collinear with *Iba4xEXLA2*, which is collinear with *Ibw6xEXLA2*, which is collinear with *Iba6xEXLA2*. Additionally, six *Itr2xEXLBs*, five *Iba4xEXLBs*, eight *Ibw6xEXLBs*, and six *Iba6xEXLBs* have one-to-one-to-one-to-one collinearity relationships. For example, *Itr2xEXLA4* is collinear with *Iba4xEXLA2*, which is collinear with *Ibw6xEXLA2*, which is collinear with *Iba6xEXLA2*. These complex collinearity relationships indicated that these four species have a very close evolutionary relationship and are consistent with recent evolutionary studies [26,29,33].

### 2.4. Phylogenetic Analysis

**Figure 3:**
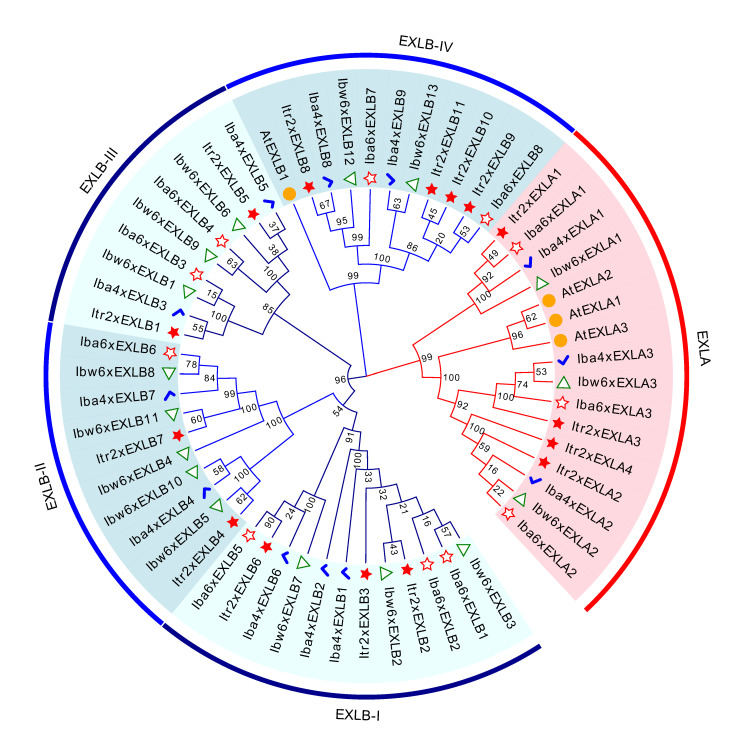
Phylogenetic tree of expansin-like proteins. The phylogenetic tree was constructed using expansin-like proteins in Y22 (Itr2x), Y428B (Iba4x), Y601 (Ibw6x), NH (Iba6x), and *A. thaliana* (At), as listed in Table S1. The EXLBs were classified into subclades EXLB-I, EXLB-II, EXLB-III, and EXLB-IV. Bootstrap values are marked on the ML tree. Genes from the same species are marked by the same symbol and color. The symbols and colors are as follows: orange dots represent *A. thaliana*; red stars represent Y22; blue checkmarks represent Y428B; green triangles represent Y601; and red hollow stars represent NH.

To explore the phylogenetic relationship of expansin-like proteins, the EXLA and EXLB protein sequences from A. thaliana, Y22, Y428B, Y601, and NH were extracted and used to construct a maximum-likelihood (ML) phylogenetic tree (Figure 3). According to the ML tree, Itr2xEXLAs, Iba4xEXLAs, Ibw6xEXLAs, Iba6xEXLAs, along with three AtEXLAs from *A. thaliana*, formed one subclade, indicating that EXLAs have a closely phylogenetical relationship among these species. The number of EXLB members was much larger than the number of EXLAs, and they could be divided into four subclades (Figure 3). Each subclade contains EXLBs from Y22, Y428B, Y601, and NH, indicating that the members within each subclade may also have a close evolutionary relationship, and that the four subclades maintain the diversity of these proteins.

### 2.5. Motifs and Gene Structure Analysis

To further evaluate the protein sequence characteristics of EXLAs and EXLBs in Y22, Y428B, Y601, and NH, we detected conserved motif composition using the MEME server system. Analysis of the EXLA subfamily revealed that the motif count ranged from 10 to 11 in Y22, and from 10 to 12 in Y428B, Y601, and NH. Motifs 3–6 and 10 were found in all EXLAs. EXLA1s, which lacks motif 13, forms a distinct minor subclade that is paraphyletic with respect to other EXLAs. Similarly, EXLA3 constitutes another minor subclade, characterized by the absence of motif 7 but the presence of motifs 11 and 19. This EXLA3 clade is paraphyletic with the minor clade comprising EXLA2 and EXLA4.

EXLBs could be further divided into four subfamilies, and motifs 5, 7, and 9 were found in all of these subfamilies. Multiple motifs were also found in the same EXLB subfamily, with some compositional differences among the subfamily members. For example, Iba4xEXLB7, Ibw6xEXLB8, and Iba6xEXLB6 have the largest number of motifs, while Iba4xEXLB6 has the smallest number of motifs. Most EXLBs contain 10–12 motifs. These results suggest that the number and composition of motifs vary observably among these EXLA and EXLB subfamilies, and that these differences may lead to distinct and diverse functions in expansin-like proteins.

**Figure 4:**
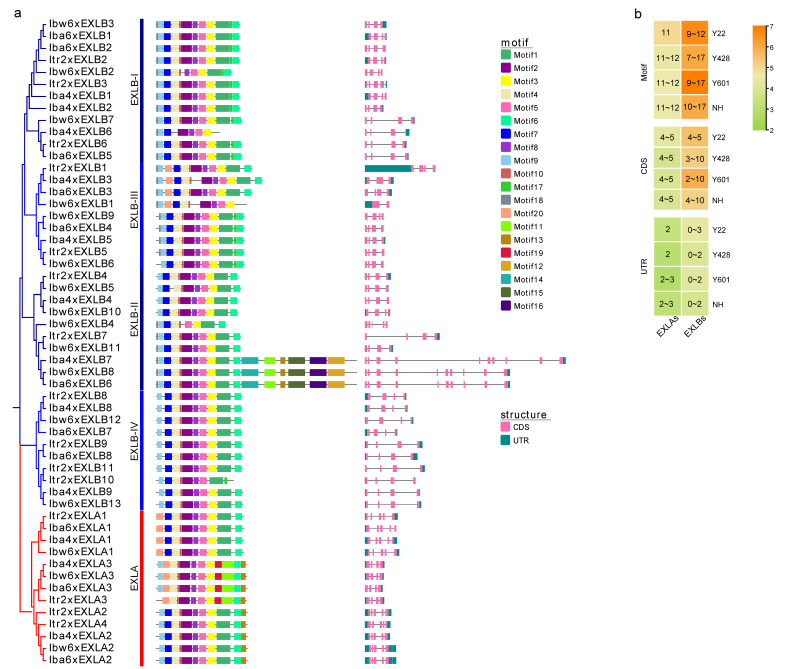
Motif compositions and gene structure of *EXLAs* and *EXLBs* in four *Ipomoea* species. (**a**) Phylogenetic tree and motif compositions of EXLAs and EXLBs. The phylogenetic tree was constructed using the maximum-likelihood (ML) method in MEGA X program with the LG + G + I model and 1000 bootstrap replications. This analysis differs from that in Figure 3, as it did not include *A. thaliana* expansin-like proteins for comparison. The conserved motifs are represented by boxes with different colors, and the motif sequences are listed in Table S4. The right part shows the gene structure organization of *EXLAs* and *EXLBs*, and the introns and exons are marked. (**b**) The variation range of motifs, and CDS and UTR in four *Ipomoea* species. The color is shown by a log scale.

An analysis of the gene structural diversity of *EXLAs* and *EXLBs* was performed. The results showed that the numbers of CDS in *EXLAs* ranged from 4 to 5, while those in EXLBs ranged from 2 to 10 (Figure 4b). The diploid *I. trifida* Y22 has the smallest range of variation in CDS number. All *EXLA* genes contain at least one UTR, whereas some *EXLBs* have no UTR. *Iba4xEXLB7* has the longest gene sequence, and *Itr2xEXLB1* has the longest UTR region. These gene structure differences between *EXLAs* and *EXLBs* may have resulted from the long-term evolution of the genome.

### 2.6. Potential Cis-Element Analysis in Gene Promoter Regions

**Figure 5:**
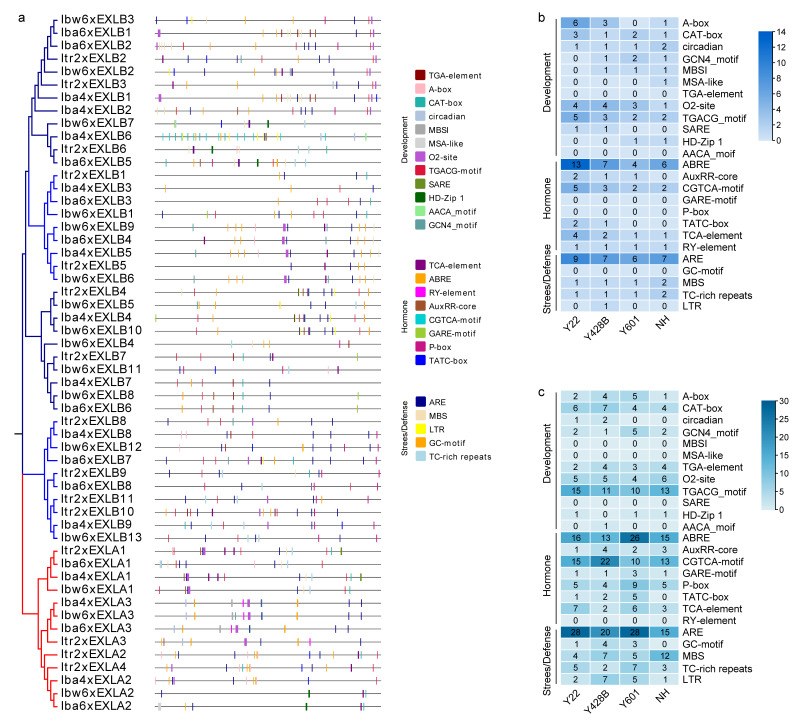
Potential cis-element prediction in the promoter regions of expansin-like genes. (**a**) ML tree and cis-element locations. Different colored symbols represent different cis-elements. (**b**,**c**) Statistical heatmaps of cis-elements in the 2000 bp upstream promoter regions of EXLAs and EXLBs, respectively. The numbers in the heatmap represent the total quantity of each element in each species.

Cis-elements are important for the regulation of gene expression and many biological processes. To explore how cis-elements in the expansin-like gene family regulate these biological processes, we used PlantCARE to predict promoter cis-elements in the 2000 bp promoter regions upstream of the expansin-like genes. The analysis identified cis-acting regulatory elements, involved in core promoter elements (CAAT-box and TATA-box), binding sites, as well as development-related, hormone-responsive, light-responsive, and defense- and stress-responsive elements. Among them, the most abundant elements were core promoter elements and light-responsive elements, followed by development-related, hormone-responsive, and defense- and stress-responsive elements (Figure 5).

Twelve development-related elements (such as, TGA-element, A-box) were found in the promoter regions of expansin-like genes (Figure 5a,b). The most abundant element in *EXLB* promoter regions was TGACG_motif, which was more abundant than in *EXLA* promoter regions. In the promoter regions, the TGA-element and AACA_motif were absent from *EXLAs* but present in *EXLBs*; conversely, MBSI, MSA-like, and SARE elements were absent from *EXLBs* but present in *EXLAs* (Figure 5b,c). For example, the AACA_motif element was found only in the *Iba4xEXLB6* promoter region of wild sweet potato Y428B, and the MSA-like element was also found only in the *Iba6xEXLA2* promoter region of cultivated sweet potato NH (Figure 5a–c).

Eight hormone-related elements and six defense- and stress-related elements were found in the promoter regions of expansin-like genes (Figure 5a–c). The most abundant hormone-responsive element in *EXLA* promoter regions was ABRE, whereas it was ABRE and CGTCA-motif in *EXLB* promoter regions. The most abundant defense- and stress-responsive element in both EXLA and EXLB promoter regions was ARE. No GARE-motif, P-box, GC-motif, and TGA-box elements that were found in the promoter region of *EXLAs*, and no RY-element was found in those of *EXLBs*; on the contrary, these elements were present in EXLB and EXLA promoter regions, respectively (Figure 5b). The TGA-box element was present in two copies and was found only in the *Iba4xEXLB6* promoter region of Y428B (Figure 5a). These results showed the differences in cis-acting element types in the 2000bp upstream promoter regions of *EXLA* and *EXLB* subfamilies. These differences may be responsible for differences in the regulation of gene expression.

### 2.7. Protein Interaction Network Analysis for Expansin-like Proteins

Exploring the interaction relationships between expansin-like proteins and other proteins could be conducive to revealing their regulatory networks, and is conducive to research on gene function and a better understanding of SR development in sweet potato. Here, we analyzed the protein interaction network of EXLA and EXLB proteins using the STRING website, based on an ortholog analysis of *A. thaliana* expansin-like proteins. The results (Figure S1) showed that no protein interactions were observed within and between EXLA and EXLB subfamilies. Integrated analysis of the protein–protein interaction network (Figure S1, based on *A. thaliana* orthologs) and phylogenetic tree (Figure 3) suggests that proteins within subclades EXLB-IV and EXLA (Figure 3) may have the potential to interact with proteins homologous to AtXTH22, AtXTH23, and AtXTH18. The XTH proteins belong to the xyloglucan endotransglucosylase/hydrolase family and are one of the key enzymes involved in the remodeling of the plant cell wall, functioning by cutting and rejoining xyloglucans [2]. These results from the protein interaction network analysis are helpful for studying cell wall development and will further facilitate research on SR development and its regulatory mechanism.

### 2.8. Expression Analysis of Expansin-like Genes During SR Development

**Figure 6:**
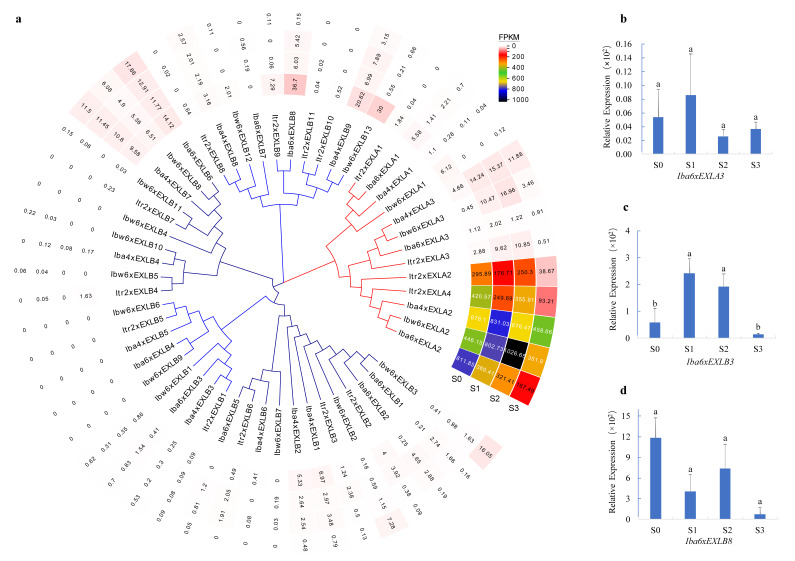
Expression patterns of expansin-like genes during SR development. (**a**) Expression values (RPKM) are shown in the heatmap, *p*-value ≤ 0.05. The inner part of the circular heatmap displays the ML tree. (**b**–**d**) Relative expression of *expansin-like* genes analyzed by qRT-PCR. The lowercase letters “a” and “b” above the histogram represent significant differences identified by one-way ANOVA followed by multiple comparisons, *p*-value ≤ 0.05.

Expansins have been reported to cause cell wall loosening and contribute to cell enlargement in different developmental processes. To explore the expression patterns and probable roles of expansin-like genes in SR development, roots at four typical stages of each species, including the stages from adventitious root (AR, S0), initiating SR (ISR, S1), young SR (YSR, S2), and mature SR (MSR, S3), were sampled for RNA-sequencing. The results showed that all the members of *EXLAs* were expressed (FPKM > 1 in at least one developmental stage) during root development (Figure 6a). Among them, *EXLA2s* and *Itr2xEXLA4* were highly expressed, while *EXLA3s* maintained relatively high expression levels during root development in wild relatives, whereas its expression was lower in sweet potato NH, and *EXLA1s* showed minimal to no detectable expression (Figure 6a, Figure S2a). We found that the protein sequence of the reported IbEXP1 is highly similar to that of EXLA2s and Itr2xEXLA4. Since *IbEXP1* plays a negative role in SR formation [22], *EXLA2s* and *Itr2xEXLA4* may play similar roles in SR development.

Among the *EXLBs*, 21 out of 41 members were expressed (FPKM > 1 in at least one developmental stage) during SR development in the four species. Most of the *EXLBs* expressed at relatively low expression level and showed a downward trend, such as *Iba6xEXLB8*, *Iba4xEXLB9*, and *Ibw6xEXLB13* (Figure 6a, Figure S2b). A small subset of EXLBs, such as *Ibw6xEXLB3* and *Iba4xEXLB7*, showed upregulated expression. We randomly selected three genes and conducted qRT-PCR analysis on root samples of NH at four different stages. The expression patterns of *Iba6xEXLA3, Iba6xEXLB3,* and *Iba6xEXLB8* were consistent with the transcriptome sequencing results (Figure 6b–d).

### 2.9. Analysis of Protein Expression Differences in SRs

#### 2.9.1. Analysis of Differentially Expressed Eukaryotic Orthologous Groups (KOGs) of Proteins

**Figure 7:**
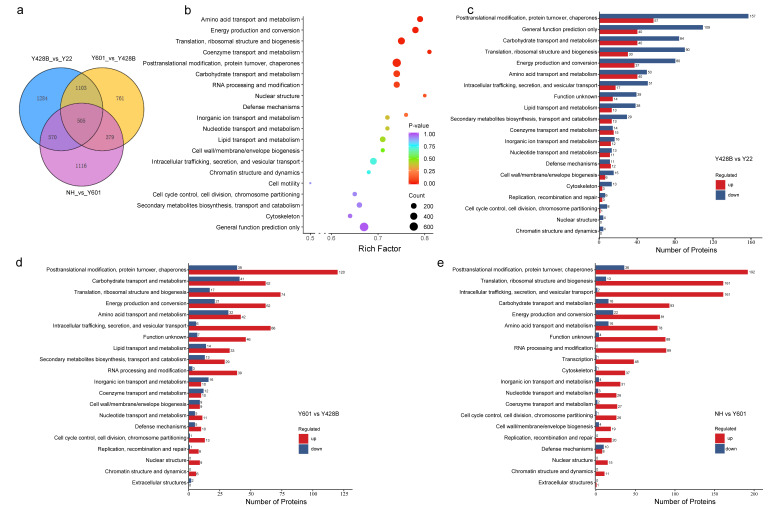
KOG analysis of differential expressed proteins identified by 4D-DIA. (**a**) Differential expressed proteins in the comparisons Y428B vs. Y22, Y601 vs. Y428B, and NH vs. Y601 are shown in the Venn diagram. (**b**) KOG annotation of differentially expressed proteins in the comparison NH vs. Y601 vs. Y22 vs. Y428B. (**c**–**e**) Number of up- and downregulated differentially expressed proteins in each KOG functional item for the comparison pairs Y428B vs. Y22 (**c**), Y601 vs. Y428B (**d**), and NH vs. Y601 (**e**), respectively. The horizontal axis represents the number of differentially expressed proteins, and the vertical axis represents the KOG functional item name. Red and blue colors represent up- and downregulated proteins, respectively; *p*-value ≤ 0.05.

In this work, the cultivated variety NH develops well-formed edible SRs, while the wild relatives Y601 and Y22 develop small SRs, and Y428B forms pencil roots. To explore the potential roles of different proteins in SR development, we sampled roots at the S3 stage from the four species for protein expression analysis using 4D-DIA (four-dimensional data independent acquisition) technology [34]. Based on the resulting proteomic data, we performed a comparative analysis of NH, Y601, Y22, and Y428B. KEGG enrichment analysis of 3000 differentially expressed proteins indicated that not only pathways related to starch, protein, and cell metabolism are involved in SR development, but also the responses and adaptations of roots to the environment (Figure S3). Through comparative analysis of differentially expressed proteins, we found that the number of common differentially expressed KOG proteins was 1608 between Y428B_vs._Y22 and Y601_vs._Y428B, and 1075 between Y428B_vs._Y22 and NH_vs._Y601. The number of common differentially expressed proteins across all the three groups (NH_vs._Y601, Y601_vs._Y428B, and Y428B_vs._Y22) was 505 (Figure 7a). We further performed differentially expressed protein analysis with KOG annotation in the four-way comparison (NH vs. Y601 vs. Y22 vs. Y428B), and found that the functional categories included ‘Cell wall/membrane/envelope biogenesis’ and ‘Cell cycle control, cell division, chromosome partitioning’. Additionally, ‘Carbohydrate transport and metabolism’ was among the top five functional categories. These results suggested that these categories may be related to the degree of root development (Figure 7b).

We further performed functional annotations on the differentially expressed proteins (Figure 7c–e) and found that in the comparison Y428B_vs._Y22, all enriched KOG categories from this analysis—including those directly related to cell wall development such as ‘Cell wall/membrane/envelope biogenesis’ and ‘Cell cycle control, cell division, chromosome partitioning’, as well as other top five enriched functional categories—showed a common trend: the numbers of downregulated proteins were significantly greater than that of upregulated proteins (Figure 7c). These results may reveal why Y428B forms pencil roots whereas Y22 forms SR, and these downregulated proteins may relate to pencil root formation. On the contrary, in the comparisons Y601_vs._Y428B and NH_vs._Y601 (Figure 7d,e), the numbers of upregulated proteins in all enriched KOG categories were much greater than that of downregulated proteins in both comparisons. As a wild sweet potato, Y601 can form small SRs, whereas the cultivated sweet potato NH forms large edible SRs. Therefore, these upregulated proteins, together with the downregulation of others, may coordinately facilitate root or small SRs swelling toward large SRs.

#### 2.9.2. Protein Expression Analysis of EXLAs and EXLBs in SR

**Figure 8:**
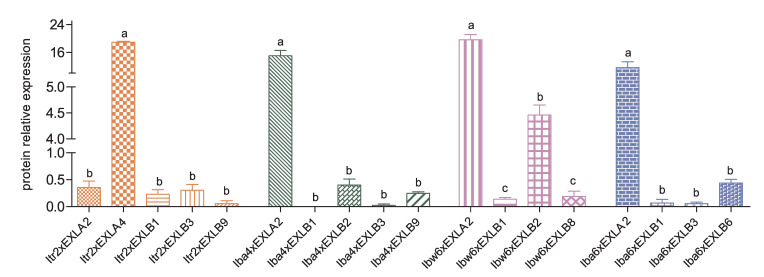
Protein expression of EXLAs and EXLBs detected by 4D-DIA. The histogram uses distinct colors for four species, with varying shapes further distinguishing genes within each species. The lowercase letters above the histogram represent significant differences identified by multiple comparisons following one-way ANOVA within each species, *p*-value ≤ 0.05.

The 4D-DIA results showed that there were 5, 4, 4, and 4 expansin-like proteins expressed in S3 stage roots of Y22, Y428B, Y601, and NH, respectively, accounting for one quarter to one third of the total expansin-like genes in each species (Figure 8). For example, *EXLA3s* were expressed in stages S0 to S3, but the corresponding proteins were not detected in stage S3 in any of the four species. This may indicate the presence of translation inhibition or rapid protein degradation. Among the expressed proteins, Itr2xEXLA4, Iba4xEXLA2, Ibw6xEXLA2, and Iba6xEXLA2 were highly expressed (Figures S3 and Figure 8). The protein sequences of Itr2xEXLA4, Iba4xEXLA2, Ibw6xEXLA2, and Iba6xEXLA2 are highly similar to the previously reported IbEXP1 [22]. The expression level of Iba6xEXLA2 in NH is relatively lower than that in the three wild relatives (Y22, Y428B, and Y601). Y22 has the longest FRSS [21], Y601 has an intermediate length [29], and NH has the shortest FRSS. This phenotype is consistent with the function of *IbEXP1*, where downregulation of *IbEXP1* shortened the FRSS and enhanced SR development in sweet potato [22]. These results not only demonstrate the evolutionary conservation of this protein sequence between wild and cultivated sweet potato, but also suggest that storage root swelling might be attributed to evolutionary changes in the regulation of the corresponding gene.

Compared with the expressed EXLAs, EXLBs were expressed at relatively low levels. Notably, the motif structures of Iba4xEXLB1 and Iba4xEXLB2 are identical, and they are adjacent in the phylogenetic tree (Figure 4). This might result in functional redundancy between these two proteins and a consequently low expression of Iba4xEXLB1, which was undetected by 4D-DIA analysis (Figure 8). Itr2xEXLB3, Iba4xEXLB2, Ibw6xEXLB2, Iba6xEXLB1, and Iba6xEXLB2 share identical motif structures and are clustered in the EXLB-I clade. In this work, the wild materials (Y22, Y601, and Y428B) develop small SRs or pencil roots, whereas the cultivated variety NH forms large and edible SRs. Our finding that Iba6xEXLB2 was not expressed/detected and that *Iba6xEXLB1* exhibits downregulated in the cultivated variety may therefore play an important role in SR swelling in sweet potato. Interestingly, Itr2xEXLA4 in Y22 and the EXLA2 homologs in Y428, Y601 and NH, showed significantly high protein expression. This pattern may indicate that these proteins are essential for promoting and maintaining storage root growth. Collectively, these results suggest that the contrasting expression patterns between EXLA and EXLB subfamilies could be a result of long-term evolution, allowing different members to assume distinct functional roles in root development.

### 2.10. Subcellular Localization of Proteins IbaEXLB2 and IbaEXLB8

**Figure 9:**
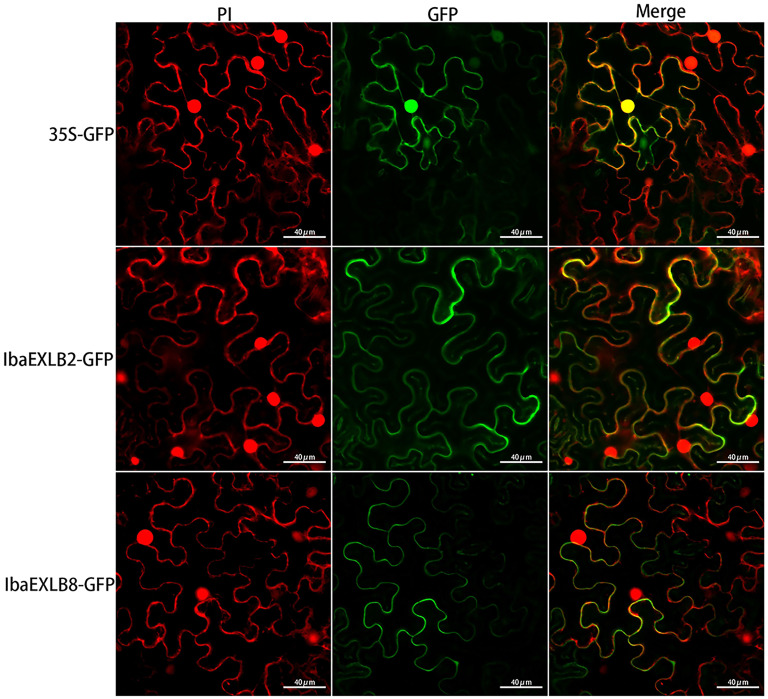
Subcellular localization of proteins IbaEXLB2 and IbaEXLB8.

Expansin is known to be a cell wall protein that promotes cell wall expansion together with other proteins such as extensin [2]. To further confirm the subcellular localization of these expansin-like proteins, we constructed the expression vectors *35S::IbaEXLB2-GFP* and *35S::IbaEXLB8-GFP*, and used *35S::GFP* as the control. We transiently expressed these constructs in tobacco epidermal cells and observed their localization using a laser scanning confocal microscope. The control *35S::GFP* signal was detected in both the nucleus and cell wall/membrane, whereas the signals from *35S::IbaEXLB2-GFP* and *35S::IbaEXLB8-GFP* were localized in the cell wall/membrane (Figure 9).

## 3. Discussion

Expansin plays a significant role in plant cell growth and development, participating in various physiological processes [35,36]. For example, *HvEXPB7* regulates root development in barley [37]; overexpression of *GsEXPB1* significantly increases the number of hairy roots, root length, and weight in soybean [13]; overexpression of *GsEXLB14* enhances the tolerance of hairy roots to salt and drought stresses in soybean [12]; and overexpression of *A. thaliana EXPA17* promotes lateral root formation in the presence of auxin [38]. Sweet potato is the seventh most important food crop worldwide, and its SR differs anatomically from those of soybean, rice, barley, and *A. thaliana*. SRs swelling is key to its yield. Sweet potato *IbEXP1* [22] and *IbEXPA4* [23] have been shown to regulate the FRSS development and thereby affect SR development. The expression levels of *IbEXP1*, *IbEXP2*, and *IbEXPL1* are significantly correlated with changes in cell wall composition in sweet potato, influencing eating quality and postharvest processing quality [39]. Recently, the expansin gene family has been reported in *I. trifida* 2x and sweet potato [20,21]. Nevertheless, the roles of EXLA and EXLB in *Ipomoea* species, particularly in wild relatives, remain poorly understood, especially in relation to SR development. Investigating genes involved in sweet potato growth and development and their potential regulatory mechanisms is crucial for enhancing yield and improving quality. Based on recent studies, cultivated sweet potato may have originated from *I. aequatoriensis* and *I. batatas* 4x [29,40], and the *I. trifida* 2x is very closely related to *I. batatas* 4x [29]. As previously reported, among the 600–700 species of *Ipomoea*, at least 63 species can form SR [25,26]; however, the ability of *I. trifida* 2x and wild *I. batatas* 6x to form SR has not been recorded. In this work, compared with Y428B, Y22 and Y601 are wild types that can form SR, making our work contributing to a better understanding of the SR evolution. Meanwhile, the ability of Y22 and Y601 to form SRs provides an important material basis for the study of SR development, especially for Y22, being a diploid with a relatively simple genome, and being an excellent material for studying gene functions. Therefore, our work focused on the structural and evolutionary characteristics of the expansin-like protein subfamily in *I. trifida* 2x, wild *I. batatas* 4x, wild *I. batatas* 6x, and cultivated sweet potato, and systematically analyzed the gene expression patterns during SR development as well as their protein expression profiles in mature SR. Our work not only provides an important theoretical foundation for understanding the molecular mechanisms of SR development but also serves as a basis for molecular breeding and genetic improvement in sweet potato.

RNA-sequencing is widely used for gene expression profiling [41,42], while proteome analysis is rare in sweet potato and its wild relatives. Proteins are the functional executors of gene function, and their expression level directly determines the strength of related functions. Therefore, building upon our transcriptomic analysis, we further conducted a comparative proteomic study. Our work may represent the first proteomic analysis focused on SR development in sweet potato wild relatives. The proteomic analysis revealed that differentially expressed proteins during SR development are significantly enriched in KOG functional categories directly pertaining to cell wall dynamics (‘Cell wall/membrane/envelope biogenesis’), carbohydrate metabolism (‘Carbohydrate transport and metabolism’), and cellular growth (‘Cell cycle control, cell division, chromosome partitioning’). Notably, through comparisons between pencil root and SR (Y428B vs. Y22), between SR with weaker swelling capacity and pencil root (Y601 vs. Y428B), and between SR with weaker swelling capacity and SR with strong swelling capacity (NH vs. Y601), we found that the up- and downregulation of proteins within these categories are closely associated with SR swelling (Figure 7). Significantly, EXLA and EXLB proteins, which are encompassed within these differentially expressed proteins, exhibited characteristic high and low expression patterns (Figure 8), respectively, and they may, even in interactions with other expansins, present a compelling complementary regulatory pattern in the hexaploid sweet potato. However, this hypothesis requires further in-depth investigation in future work.

## 4. Materials and Methods

### 4.1. Materials

The plant materials used in this study included Y22, an *I. trifida* 2x strain with accession number CIP 698014 [19]; Y428B, a wild *I. batatas* 4x multiple hybrid strain with accession number CIP 695150B [29]; Y601, a wild *I. batatas* 6x strain [29]; and the cultivated *I. batatas* 6x variety Nancy Hall (NH). The genomes of these four materials were assembled by our group and can be obtained from NGDC (https://ngdc.cncb.ac.cn/). The accessions are PRJCA015454, PRJCA015460, and PRJCA054709, respectively.

### 4.2. Identification and Characterization of Expansin-like Family

The protein sequences of Y22, Y428B, Y601, and NH were scanned as queries for BLASTP searches (e-value < 1 × 10^−5^) against *A. thaliana* protein sequences obtained from TAIR (https://www.arabidopsis.org/, (accessed on 24 March 2024)). Protein characteristics were calculated using TBtools-II v2.315 [31] with default parameters.

### 4.3. Chromosomal Localization and Collinearity Analysis

The expansin-like genes of Y22, Y428B, Y601, and NH were located and mapped to their respective chromosomes using TBtools-II v2.315 (default parameters) [31] according to their genome GFF3 files. Gene duplication patterns and interspecies collinearity were analyzed using the One step MCScanX plugin in TBtools-II v2.315 (default parameters) [31].

### 4.4. Phylogenetic Analysis

The expansin-like proteins from Y22, Y428B, Y601, NH, and *A. thaliana* were used for phylogenetic analysis. The maximum-likelihood (ML) phylogenetic tree was constructed using MEGA X program with model (LG + G + I) and 1000 bootstrap replications, and visualized with the Evolview v3 [43].

### 4.5. Protein Domains and Gene Structure Analysis

Protein domains were predicted using MEME (v5.5.8) (https://meme-suite.org/meme/tools/meme (accessed on 27 September 2024)). The GFF3 information of expansin-like genes was extracted using a Perl script. Potential cis-elements in the 2000 bp upstream region of the expansin-like genes were predicted using PlantCARE (https://bioinformatics.psb.ugent.be/webtools/plantcare/html/ (accessed on 27 September 2024)) [44] and visualized with Evolview v3 [43].

### 4.6. Protein Interaction Network Analysis for Expansin-like Proteins

Based on the homologous proteins from *A. thaliana*, we used STRING to predict the interaction relationships between expansin-like proteins and between expansin-like proteins and other proteins [45].

### 4.7. Gene Expression Analysis During SR Development

Plants of Y22, Y428B, Y601, and NH were grown in the same experimental field and sampled simultaneously. AR, ISR, YSR, and MSR were collected at 105 days after planting, with sampling having three repeats based on root phenotypes as described previously [19]. Each sample was grinded in liquid nitrogen, then aliquoted into EP tubes and stored at −80 °C for transcriptome sequencing, qRT-PCR, and proteome analysis. Total RNA for RNA-seq and qRT-PCR was extracted using TRIzol reagent (Invitrogen, Waltham, MA, USA) and treated with RNase-free DNase I (Promega, Madison, WI, USA). Library construction, RNA-sequencing, and transcriptome analysis were performed as described previously [21]. Differentially expressed genes were identified using the thresholds of an adjusted *p*-value ≤ 0.05.

Total RNA was reverse transcribed using HiScript ^®^II 1st Strand cDNA Synthesis Kit (+gDNA wiper) (R212, Vazyme, Nanjing, China) according to the manufacturer’s instructions. qRT-PCR was performed using a Quanti Nova SYBR Green PCR Kit (500, Qiagen I Sidbio, Chongqing, China) on a Roche LightCycler 96 according to the manufacturer’s instructions. The PCR conditions were 5 min at 95 °C, followed by 40 cycles of 10 s at 95 °C, 10 s at 58 °C, and 10 s at 72 °C, with three repeats. The *I. batatas* Actin gene was used as an internal control. Primer pairs for selected genes are listed in Table S5. The 2^–ΔΔCT^ method was used to calculate relative gene expression [46].

### 4.8. Protein Extraction and 4D-DIA Proteome Sequencing

S3 stage root samples from Y22, Y428B, Y601, and NH were used for proteomic sequencing. Proteins were extracted using the acetone precipitation method, and their concentration was determined with a BCA kit (Biyuntian, Chongqing, China) according to the manufacturer’s instructions. Equal amounts of proteins from each sample were digested with trypsin, then concentrated by vacuum centrifugation and redissolved in 0.1% (*v*/*v*) formic acid. Approximately 200 ng peptides were subjected to liquid chromatography (LC) on a nanoElute UHPLC (Bruker Daltonics, Bremen, Germany). Mass spectrometric (MS) data were collected using DDA-PASEF and DIA-PASEF modes on the timsTOF Pro2 (Bruker Daltonics, Bremen, Germany). MS raw data were analyzed using DIA-NN (v1.8.1) with the library-free method. The protein annotation files for Y22 (32,445 sequences), Y428B (33,122 sequences), Y601 (35,099 sequences), and NH (34,170 sequences) were used to create a spectra library via deep learning algorithms of neural networks. The false discovery rate (FDR) was adjusted to <1% at both protein and precursor ion levels. Proteins were considered confidently identified and used for subsequent quantification only if they were supported by at least two unique peptides, with a peptide length of ≥7 amino acids, and passed this FDR threshold. To functionally characterize the identified proteins, we conducted functional annotations for all identified proteins and for differentially expressed proteins (*p*-value ≤ 0.05) between Y22, Y428B, Y601, and NH, including gene ontology (GO), KOG functional classification, KEGG pathways, and protein domain analyses. Protein extraction, detection, and quantitative profiling were performed by Wuhan Metware Biotechnology Co., Ltd. (Wuhan, China) (www.metware.cn).

### 4.9. Subcellular Localization of Expansin-like Proteins

The 35S::*IbaEXLB2*-GFP and 35S::*IbaEXLB8*-GFP expression vectors were constructed using the In-Fusion method. Both constructs were transformed into *Agrobacterium tumefaciens* GV3101 via the liquid nitrogen freeze–thaw method. Bacterial strain cultures were grown to an OD600 of 0.5–0.6, harvested, and resuspended for transient transformation of *Nicotiana benthamiana* epidermal cells. After transformation, tobacco plants were kept in a dark environment at 21 °C for 24 h, then transferred to normal growth conditions for another 24–48 h. Before imaging, leaf samples were incubated for 30 min at room temperature in a propidium iodide (PI) working solution (0.5 mg/L). Fluorescence signals in transformed leaves were visualized using a confocal microscope (Olympus, Tokyo, Japan).

## Data Availability

The proteomic data have been deposited in NGDC (https://ngdc.cncb.ac.cn/) under accession number PRJCA054710, and the genome data accessions were PRJCA015454, PRJCA015460, and PRJCA054709, respectively. The transcriptomic data are available from the corresponding author upon request.

## Supplementary Materials

The following supporting information can be downloaded at https://www.mdpi.com/article/10.3390/plants15020305/s1; Figure S1: Potential interaction network of EXLAs and EXLBs based on *A. thaliana* orthologs; Figure S2: Analysis of gene expression patterns; Figure S3. KEGG pathway analysis of differentially expressed proteins from the comparative analysis of NH, Y601, Y22, and Y428B by 4D-DIA. Table S1: Details of the expansin-like proteins in Y22, Y428B, Y601, and NH; Table S2: The duplication type of expansin-like genes in Y22, Y428B, Y601 and NH; Table S3: The one-match-one gene collinearity relationships among four genomes; Table S4: The protein sequences of different conserved motifs; Table S5: The primer pairs of selected genes for qRT-PCR.

## References

[1] McQueen-Mason S., Durachko D.M., Cosgrove D.J. (1992). Two Endogenous Proteins That Induce Cell Wall Extension in Plants. Plant Cell.

[2] Cosgrove D.J. (2005). Growth of the plant cell wall. Nat. Rev. Mol. Cell Biol..

[3] Sampedro J., Cosgrove D.J. (2005). The expansin superfamily. Genome Biol..

[4] Zhang W., Yan H.W., Chen W.J., Liu J.Y., Jiang C.P., Jiang H.Y., Zhu S.W., Cheng B.J. (2014). Genome-wide identification and characterization of maize expansin genes expressed in endosperm. Mol. Genet. Genom..

[5] Han Z.S., Liu Y.L., Deng X., Liu D.M., Liu Y., Hu Y.K., Yan Y.M. (2019). Genome-wide identification and expression analysis of expansin gene family in common wheat (*Triticum aestivum* L.). BMC Genom..

[6] Zhu Y., Wu N.N., Song W.L., Yin G.J., Qin Y.J., Yan Y.M., Hu Y.K. (2014). Soybean (*Glycine max*) expansin gene superfamily origins: Segmental and tandem duplication events followed by divergent selection among subfamilies. BMC Plant Biol..

[7] Lv L.M., Zuo D.Y., Wang X.F., Cheng H.L., Zhang Y.P., Wang Q.L., Song G.L., Ma Z.Y. (2020). Genome-wide identification of the expansin gene family reveals that genes are involved in fibre cell growth in cotton. BMC Plant Biol..

[8] Zenoni S., Fasoli M., Tornielli G.B., Dal Santo S., Sanson A., de Groot P., Sordo S., Citterio S., Monti F., Pezzotti M. (2011). Overexpression of *PhEXPA1* increases cell size, modifies cell wall polymer composition and affects the timing of axillary meristem development in Petunia hybrida. New Phytol..

[9] Che J., Yamaji N., Shen R.F., Ma J.F. (2016). An Al-inducible expansin gene, *OsEXPA10* is involved in root cell elongation of rice. Plant J. For. Cell Mol. Biol..

[10] Yang J., Zhang G., An J., Li Q., Chen Y., Zhao X., Wu J., Wang Y., Hao Q., Wang W. (2020). Expansin gene *TaEXPA2* positively regulates drought tolerance in transgenic wheat (*Triticum aestivum* L.). Plant Sci..

[11] Wang X., Ma J., He F., Wang L., Zhang T., Liu D., Xu Y., Li F., Feng X. (2024). A Study on the Functional Identification of Overexpressing Winter Wheat Expansin Gene *TaEXPA7-B* in Rice under Salt Stress. Int. J. Mol. Sci..

[12] Wang L., Zhang T., Li C., Zhou C., Liu B., Wu Y., He F., Xu Y., Li F., Feng X. (2024). Overexpression of Wild Soybean Expansin Gene *GsEXLB14* Enhanced the Tolerance of Transgenic Soybean Hairy Roots to Salt and Drought Stresses. Plants.

[13] Feng X., Li C., He F., Xu Y., Li L., Wang X., Chen Q., Li F. (2022). Genome-Wide Identification of Expansin Genes in Wild Soybean (*Glycine soja*) and Functional Characterization of Expansin B1 (*GsEXPB1*) in Soybean Hair Root. Int. J. Mol. Sci..

[14] Liu J., Wang Y., Yang L., Wang X., Zhang J., Xu J. (2024). Characterization and functional analysis of the *PtEXLA1* gene from poplar. Plant Biotechnol. Rep..

[15] Dong B., Wang Q., Zhou D., Wang Y., Miao Y., Zhong S., Fang Q., Yang L., Xiao Z., Zhao H. (2024). Abiotic stress treatment reveals expansin like A gene *OfEXLA1* improving salt and drought tolerance of *Osmanthus fragrans* by responding to abscisic acid. Hortic. Plant J..

[16] Chen Y.-h., Xie B., An X.-h., Ma R.-p., Zhao D.-y., Cheng C.-g., Li E.-m., Zhou J.-t., Kang G.-d., Zhang Y.-z. (2022). Overexpression of the apple expansin-like gene *MdEXLB1* accelerates the softening of fruit texture in tomato. J. Integr. Agric..

[17] Cho H.T., Cosgrove D.J. (2002). Regulation of root hair initiation and expansin gene expression in *Arabidopsis*. Plant Cell.

[18] Tanaka M. (2016). Recent Progress in Molecular Studies on Storage Root Formation in Sweetpotato (*Ipomoea batatas*). Jpn. Agric. Res. Q..

[19] Li M., Yang S., Xu W., Pu Z., Feng J., Wang Z., Zhang C., Peng M., Du C., Lin F. (2019). The wild sweetpotato (*Ipomoea trifida*) genome provides insights into storage root development. BMC Plant Biol..

[20] Zhang J., Dong T., Zhu M., Du D., Liu R., Yu Q., Sun Y., Zhang Z. (2024). Transcriptome- and genome-wide systematic identification of expansin gene family and their expression in tuberous root development and stress responses in sweetpotato (*Ipomoea batatas*). Front. Plant Sci..

[21] Li M., Chen L., Lang T., Qu H., Zhang C., Feng J., Pu Z., Peng M., Lin H. (2022). Genome-Wide Identification and Expression Analysis of Expansin Gene Family in the Storage Root Development of Diploid Wild Sweetpotato *Ipomoea trifida*. Genes.

[22] Noh S.A., Lee H.S., Kim Y.S., Paek K.H., Shin J.S., Bae J.M. (2013). Down-regulation of the *IbEXP1* gene enhanced storage root development in sweetpotato. J. Exp. Bot..

[23] Zhang X., Tang C., Jiang B., Zhang R., Li M., Wu Y., Yao Z., Huang L., Luo Z., Zou H. (2024). Refining polyploid breeding in sweet potato through allele dosage enhancement. Nat. Plants.

[24] Yan M., Nie H., Wang Y., Wang X., Jarret R., Zhao J., Wang H., Yang J. (2022). Exploring and exploiting genetics and genomics for sweetpotato improvement: Status and perspectives. Plant Commun..

[25] Munoz-Rodriguez P., Carruthers T., Wood J.R.I., Williams B.R.M., Weitemier K., Kronmiller B., Goodwin Z., Sumadijaya A., Anglin N.L., Filer D. (2019). A taxonomic monograph of Ipomoea integrated across phylogenetic scales. Nat. Plants.

[26] Munoz-Rodriguez P., Carruthers T., Wood J.R.I., Williams B.R.M., Weitemier K., Kronmiller B., Ellis D., Anglin N.L., Longway L., Harris S.A. (2018). Reconciling Conflicting Phylogenies in the Origin of Sweet Potato and Dispersal to Polynesia. Curr. Biol..

[27] Kyndt T., Quispe D., Zhai H., Jarret R., Ghislain M., Liu Q., Gheysen G., Kreuze J.F. (2015). The genome of cultivated sweet potato contains Agrobacterium T-DNAs with expressed genes: An example of a naturally transgenic food crop. Proc. Natl. Acad. Sci. USA.

[28] Quispe-Huamanquispe D.G., Gheysen G., Yang J., Jarret R., Rossel G., Kreuze J.F. (2019). The horizontal gene transfer of Agrobacterium T-DNAs into the series Batatas (Genus *Ipomoea*) genome is not confined to hexaploid sweetpotato. Sci. Rep..

[29] Yan M., Li M., Wang Y., Wang X., Moeinzadeh M.H., Quispe-Huamanquispe D.G., Fan W., Fang Y., Wang Y., Nie H. (2024). Haplotype-based phylogenetic analysis and population genomics uncover the origin and domestication of sweetpotato. Mol. Plant.

[30] Yang J., Moeinzadeh M.H., Kuhl H., Helmuth J., Xiao P., Haas S., Liu G., Zheng J., Sun Z., Fan W. (2017). Haplotype-resolved sweet potato genome traces back its hexaploidization history. Nat. Plants.

[31] Chen C., Wu Y., Li J., Wang X., Zeng Z., Xu J., Liu Y., Feng J., Chen H., He Y. (2023). TBtools-II: A “one for all, all for one” bioinformatics platform for biological big-data mining. Mol. Plant.

[32] Wang Y., Tang H., Debarry J.D., Tan X., Li J., Wang X., Lee T.H., Jin H., Marler B., Guo H. (2012). MCScanX: A toolkit for detection and evolutionary analysis of gene synteny and collinearity. Nucleic Acids Res..

[33] Feng J.Y., Li M., Zhao S., Zhang C., Yang S.T., Qiao S., Tan W.F., Qu H.J., Wang D.Y., Pu Z.G. (2018). Analysis of evolution and genetic diversity of sweetpotato and its related different polyploidy wild species *I. trifida* using RAD-seq. BMC Plant Biol..

[34] Xu C., Wang L., Yin J., Qi L., Feng Y., Ji Y., Li Y., Chang S., Yuan P., Zhang Z. (2024). Wild relatives and new breeding techniques sustain Fe and Zn biofortified crop farming systems under climate change and emergencies. Cogent Food Agric..

[35] Li Y., Li B., Pang Q., Lou Y., Wang D., Wang Z. (2024). Identification and expression analysis of expansin gene family in *Salvia miltiorrhiza*. Chin. Med..

[36] Li Y., Jones L., McQueen-Mason S. (2003). Expansins and cell growth. Curr. Opin. Plant Biol..

[37] He X., Zeng J., Cao F., Ahmed I.M., Zhang G., Vincze E., Wu F. (2015). *HvEXPB7*, a novel beta-expansin gene revealed by the root hair transcriptome of Tibetan wild barley, improves root hair growth under drought stress. J. Exp. Bot..

[38] Lee H.W., Kim J. (2013). *EXPANSINA17* up-regulated by LBD18/ASL20 promotes lateral root formation during the auxin response. Plant Cell Physiol..

[39] Dong W., Li L., Cao R., Xu S., Cheng L., Yu M., Lv Z., Lu G. (2020). Changes in cell wall components and polysaccharide-degrading enzymes in relation to differences in texture during sweetpotato storage root growth. J. Plant Physiol..

[40] Wu S., Sun H., Zhao X., Hamilton J.P., Mollinari M., Gesteira G.D.S., Kitavi M., Yan M., Wang H., Yang J. (2025). Phased chromosome-level assembly provides insight into the genome architecture of hexaploid sweetpotato. Nat. Plants.

[41] Ponniah S.K., Thimmapuram J., Bhide K., Kalavacharla V., Manoharan M. (2017). Comparative analysis of the root transcriptomes of cultivated sweetpotato (*Ipomoea batatas* [L.] Lam) and its wild ancestor (*Ipomoea trifida* [Kunth] G. Don). BMC Plant Biol..

[42] Zhang K., Wu Z., Tang D., Luo K., Lu H., Liu Y., Dong J., Wang X., Lv C., Wang J. (2017). Comparative Transcriptome Analysis Reveals Critical Function of Sucrose Metabolism Related-Enzymes in Starch Accumulation in the Storage Root of Sweet Potato. Front. Plant Sci..

[43] Subramanian B., Gao S., Lercher M.J., Hu S., Chen W.H. (2019). Evolview v3: A webserver for visualization, annotation, and management of phylogenetic trees. Nucleic Acids Res..

[44] Lescot M., Dehais P., Thijs G., Marchal K., Moreau Y., Van de Peer Y., Rouze P., Rombauts S. (2002). PlantCARE, a database of plant cis-acting regulatory elements and a portal to tools for in silico analysis of promoter sequences. Nucleic Acids Res..

[45] Szklarczyk D., Gable A.L., Nastou K.C., Lyon D., Kirsch R., Pyysalo S., Doncheva N.T., Legeay M., Fang T., Bork P. (2021). The STRING database in 2021: Customizable protein-protein networks, and functional characterization of user-uploaded gene/measurement sets. Nucleic Acids Res..

[46] Livak K.J., Schmittgen T.D. (2001). Analysis of relative gene expression data using real-time quantitative PCR and the 2(-Delta Delta C(T)) Method. Methods.

