# Supplementary material for: Identification, Transcriptome, and Proteome Analysis of Expansin-like Subfamilies in the Storage Root Across I. trifida (2x), Wild (4x, 6x) and Cultivated Sweet Potatoes
Source: Plants (Basel). 2026 Jan 20;15(2):305. doi: 10.3390/plants15020305 (PMC12845480; doi:10.3390/plants15020305)
Supplement: Supplementary file 1 [file plants-15-00305-s001.zip › plants-3973866-supplementary.pdf]

Identification, Transcriptome and Proteome Analysis of Expansin-like Subfamilies in the Storage Root across *I. trifida* (2x), Wild (4x, 6x) and Cultivated Sweetpotatoes

Jingjing Li[1], Zhiyu Zhang[1], Qiuzhuo Li[2], Chunli Geng[1], Haoxi Huang[1], Xiaojian Qin[1], Yongshu Liang[1], Wenbin Nan[1], Hanma Zhang[1], Yufan Fu[3], Ming Li[1]*

[1] Chongqing Key Laboratory of Plant Environmental Adaptation Biology, College of Life Sciences, Chongqing Normal University, Chongqing 401331, China; 15012064421@163.com (J.L.); m15911789910@163.com (Z.Z); 15924846627@163.com (C.G.); 18983350449@163.com (H.H.); qinxiaojian@cqnu.edu.cn (X.Q.); yongshu-liang@yeah.net (Y.L.); nanwenbin513@163.com(W.N.); hanmazhang@126.com(H.Z.)

[2] Chongqing Three Gorges Academy of Agricultural Sciences, Wanzhou 404155, China; lqz380@163.com

[3] Engineering and Technology Research Center for Sweetpotato of Chongqing, School of Life Science, Southwest University, Chongqing 400715, China; xsfanyufu@126.com

* Correspondence: lmww1981@cqnu.edu.cn

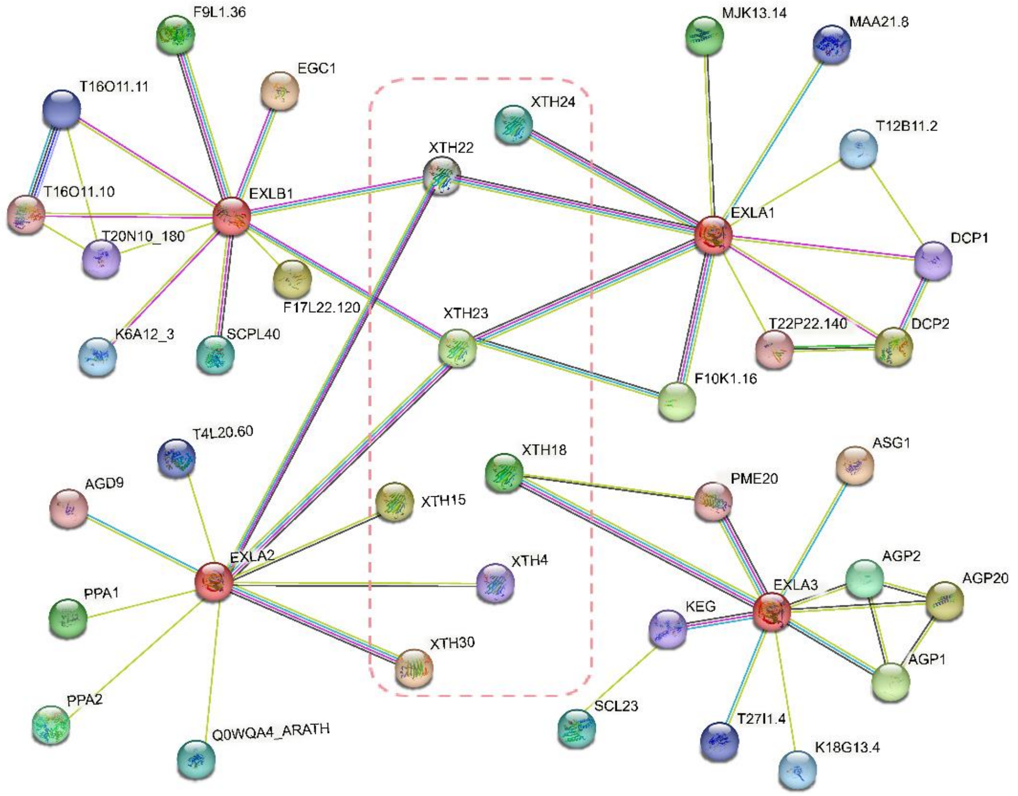

Figure S1. Potential interaction network of EXLAs and EXLBs based on *A. thaliana* orthologs.

The red dotted box shows the different XTH proteins.

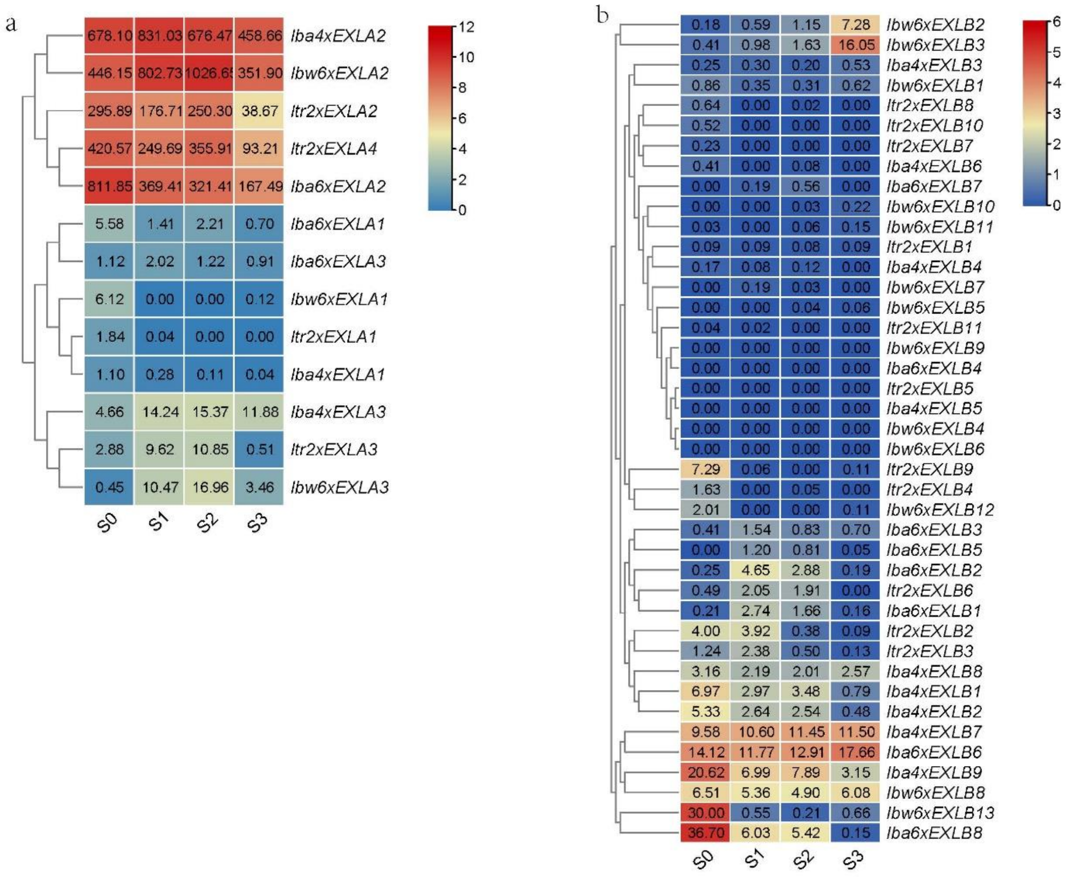

Figure S2. Analysis of gene expression patterns.

(**a**) Expression pattern of EXLA based on transcriptome analysis; (**b**) Expression pattern of EXLB based on transcriptome analysis. The values in the heatmap cells represent gene expression levels in FPKM, and the color scale is presented on a log scale.

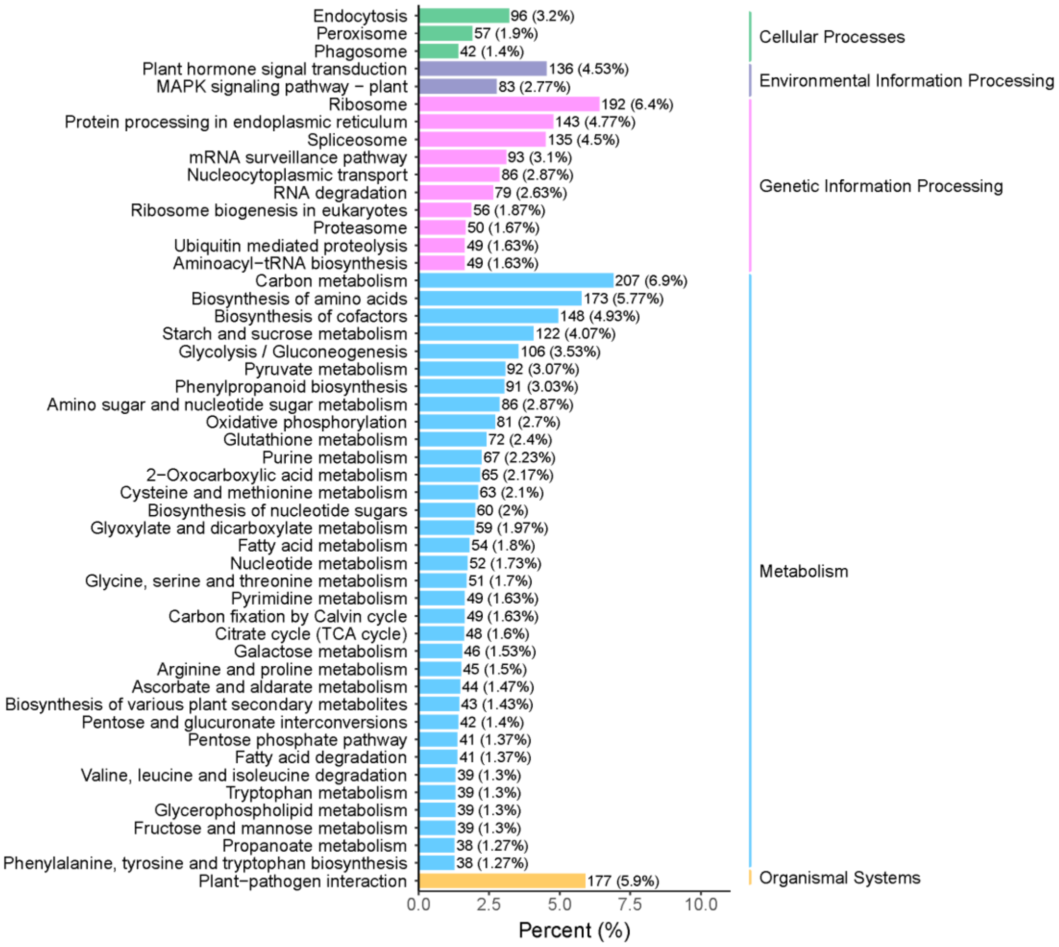

Figure S3. KEGG pathway analysis of differentially expressed proteins from the comparative analysis of NH, Y601, Y22, and Y428B by 4D-DIA.

Table S1. Details of the *expansin-like* proteins in Y22, Y428B, Y601, and NH.

Protein name	Sequence ID	Amino acid no.	Molecular weight (kD)	Theoretical pI	Instability Index	Aliphatic Index	Grand Average of Hydropathicity
Itr2xEXLA1	Itr2xGene007907	258	27.46	4.62	35.61	70.70	-0.152
Itr2xEXLA2	Itr2xGene010165	268	29.41	6.96	33.87	75.71	-0.127
Itr2xEXLA3	Itr2xGene017469	265	28.57	5.13	41.39	80.60	0.036
Itr2xEXLA4	Itr2xGene023002	267	29.20	6.91	34.37	74.53	-0.101
Iba4xEXLA1	Iba4xGene008609	258	27.50	4.57	37.38	70.70	-0.164
Iba4xEXLA2	Iba4xGene010235	268	29.41	6.96	33.87	75.71	-0.127
Iba4xEXLA3	Iba4xGene018471	266	28.61	5.13	42.79	82.52	0.065
Ibw6xEXLA1	Ibw6xGene009153	258	27.52	4.48	35.01	70.70	-0.151
Ibw6xEXLA2	Ibw6xGene010008	268	29.41	6.96	33.87	75.71	-0.127
Ibw6xEXLA3	Ibw6xGene020011	266	28.64	5.13	42.87	82.14	0.052
Iba6xEXLA1	Iba6xGene008903	258	27.38	4.60	36.81	70.70	-0.131
Iba6xEXLA2	Iba6xGene010677	268	29.41	6.96	33.87	75.71	-0.127
Iba6xEXLA3	Iba6xGene019885	266	28.62	5.29	42.04	82.89	0.073
Itr2xEXLB1	Itr2xGene007882	281	29.26	6.58	41.85	72.60	-0.088
Itr2xEXLB2	Itr2xGene007885	246	26.97	8.61	33.55	84.51	-0.165
Itr2xEXLB3	Itr2xGene007886	246	27.01	8.73	29.59	82.93	-0.146
Itr2xEXLB4	Itr2xGene021540	238	25.49	5.71	27.69	74.92	-0.108
Itr2xEXLB5	Itr2xGene021541	259	28.97	6.73	33.18	77.64	-0.183
Itr2xEXLB6	Itr2xGene021572	251	27.40	8.48	21.84	82.43	-0.096
Itr2xEXLB7	Itr2xGene021707	248	26.56	8.96	25.68	84.11	0.039
Itr2xEXLB8	Itr2xGene021868	252	28.31	5.74	24.01	79.25	-0.234
Itr2xEXLB9	Itr2xGene030545	253	28.25	6.14	21.62	78.58	-0.208
Itr2xEXLB10	Itr2xGene030548	228	25.25	6.71	22.80	77.32	-0.196
Itr2xEXLB11	Itr2xGene030554	253	28.28	6.14	22.32	78.58	-0.217
Iba4xEXLB1	Iba4xGene008624	246	26.94	8.73	30.59	82.52	-0.130
Iba4xEXLB2	Iba4xGene008625	246	27.05	8.73	27.40	81.34	-0.174
Iba4xEXLB3	Iba4xGene008626	312	32.81	5.94	42.53	70.99	-0.134
Iba4xEXLB4	Iba4xGene021760	239	25.71	6.13	32.84	74.60	-0.129
Iba4xEXLB5	Iba4xGene021763	259	28.95	6.73	32.60	77.64	-0.183
Iba4xEXLB6	Iba4xGene021791	186	20.69	8.57	37.16	92.26	0.133
Iba4xEXLB7	Iba4xGene021909	588	65.79	5.55	34.14	76.41	-0.300
Iba4xEXLB8	Iba4xGene022096	252	28.41	5.74	23.44	77.70	-0.267
Iba4xEXLB9	Iba4xGene030872	254	28.41	6.14	20.75	79.80	-0.232
Ibw6xEXLB1	Ibw6xGene009135	266	28.2	5.92	44.12	68.95	-0.176
Ibw6xEXLB2	Ibw6xGene009136	222	24.55	8.90	35.16	80.50	-0.186
Ibw6xEXLB3	Ibw6xGene009137	246	26.98	8.46	30.63	82.11	-0.179
Ibw6xEXLB4	Ibw6xGene024171	205	22.93	9.17	24.11	80.29	-0.048
Ibw6xEXLB5	Ibw6xGene024176	248	26.94	5.71	31.63	76.61	-0.073
Ibw6xEXLB6	Ibw6xGene024179	259	28.96	6.73	31.71	79.54	-0.171

Ibw6xEXLB7	Ibw6xGene024223	251	27.43	8.63	20.47	80.88	-0.089
Ibw6xEXLB8	Ibw6xGene024306	588	65.76	5.54	34.04	75.77	-0.310

Continued Table S1. Details of the *expansin-like* proteins in Y22, Y428B, Y601, and NH.

Protein name	Sequence ID	Amino acid no.	Molecular weight (kD)	Theoretical pI	Instability Index	Aliphatic Index	Grand Average of Hydropathicity
Ibw6xEXLB9	Ibw6xGene024471	259	28.98	6.73	33.18	77.64	-0.193
Ibw6xEXLB10	Ibw6xGene024474	239	25.69	6.13	33.64	74.60	-0.143
Ibw6xEXLB11	Ibw6xGene024546	248	26.73	8.96	25.22	80.56	-0.015
Ibw6xEXLB12	Ibw6xGene024700	252	28.30	5.74	23.56	78.10	-0.254
Ibw6xEXLB13	Ibw6xGene033040	254	28.39	6.14	21.05	79.80	-0.232
Iba6xEXLB1	Iba6xGene008923	246	27.05	8.61	33.20	80.12	-0.193
Iba6xEXLB2	Iba6xGene008924	246	26.97	8.93	29.90	82.11	-0.178
Iba6xEXLB3	Iba6xGene008925	281	29.22	6.14	39.00	72.60	-0.083
Iba6xEXLB4	Iba6xGene023116	259	28.95	6.73	32.15	77.64	-0.192
Iba6xEXLB5	Iba6xGene023145	251	27.40	8.48	22.18	82.03	-0.098
Iba6xEXLB6	Iba6xGene023234	588	65.76	5.48	34.54	75.60	-0.319
Iba6xEXLB7	Iba6xGene023401	252	28.26	5.74	24.49	79.64	-0.244
Iba6xEXLB8	Iba6xGene032016	253	28.27	6.14	21.41	78.97	-0.223

Table S2. The duplication type of expansin-like genes in Y22, Y428B, Y601 and NH.

Gene name	Gene ID	Corresponding gene	Duplication type
Itr2xEXLA2	Itr2xGene010165.t01	Itr2xGene023002.t01	WGD or Segmental
Itr2xEXLA4	Itr2xGene023002.t01		WGD or Segmental
Itr2xEXLB2	Itr2xGene007885.t01		Tandem
Itr2xEXLB3	Itr2xGene007886.t01		Tandem
Itr2xEXLB4	Itr2xGene021540.t01	Itr2xGene021707.t01	WGD or Segmental
Itr2xEXLB5	Itr2xGene021541.t01		Tandem
Itr2xEXLB6	Itr2xGene021572.t01	Itr2xGene021707.t01	WGD or Segmental
Itr2xEXLB7	Itr2xGene021707.t01		WGD or Segmental
Iba4xEXLB1	Iba4xGene008624.t01	Iba4xGene021760.t01<br>Iba4xGene021791.t01	WGD or Segmental
Iba4xEXLB2	Iba4xGene008625.t01		Tandem
Iba4xEXLB3	Iba4xGene008626.t01		Tandem
Iba4xEXLB4	Iba4xGene021760.t01		WGD or Segmental
Iba4xEXLB6	Iba4xGene021791.t01	Iba4xGene021909.t01	WGD or Segmental
Iba4xEXLB7	Iba4xGene021909.t01		WGD or Segmental
Ibw6xEXLB2		Ibw6xGene009136.t01	Tandem
Ibw6xEXLB3		Ibw6xGene009137.t01	Tandem
Ibw6xEXLB7	Ibw6xGene009135.t02	Ibw6xGene024223.t01	WGD or Segmental
Ibw6xEXLB9	Ibw6xGene024471.t01	Ibw6xGene024700.t01	WGD or Segmental
Ibw6xEXLB11	Ibw6xGene024306.t02	Ibw6xGene024546.t01	WGD or Segmental
Ibw6xEXLB12	Ibw6xGene024471.t01	Ibw6xGene024700.t01	WGD or Segmental
Ibw6xEXLA1	Ibw6xGene009153.t01	Ibw6xGene024546.t01	WGD or Segmental
Iba6xEXLB1	Iba6xGene008923.t01	Iba6xGene023145.t01	WGD or Segmental
Iba6xEXLB2	Iba6xGene008924.t01		Tandem
Iba6xEXLB3	Iba6xGene008925.t01		Tandem
Iba6xEXLB5	Iba6xGene023145.t01		WGD or Segmental

Table S3. The one-match-one gene collinearity relationships among four genomes.

Chromosome	One-match-one gene collinearity		Chromosome
Itr2xChr04	Itr2xEXLA2	Iba4xEXLA2	Iba4xChr04
Itr2xChr08	Itr2xEXLA3	Iba4xEXLA3	Iba4xChr08
Itr2xChr11	Itr2xEXLA4	Iba4xEXLA2	Iba4xChr04
Itr2xChr04	Itr2xEXLB1	Iba4xEXLB1	Iba4xChr04
Itr2xChr04	Itr2xEXLB1	Iba4xEXLB6	Iba4xChr10
Itr2xChr10	Itr2xEXLB4	Iba4xEXLB4	Iba4xChr10
Itr2xChr10	Itr2xEXLB6	Iba4xEXLB6	Iba4xChr10
Itr2xChr10	Itr2xEXLB7	Iba4xEXLB7	Iba4xChr10
Itr2xChr10	Itr2xEXLB8	Iba4xEXLB8	Iba4xChr10
Itr2xChr14	Itr2xEXLB9	Iba4xEXLB9	Iba4xChr14
Itr2xChr04	Itr2xEXLA1	Iba4xEXLA1	Iba4xChr04
Iba4xChr04	Iba4xEXLA2	Ibw6xEXLA2	Ibw6xChr04
Iba4xChr08	Iba4xEXLA3	Ibw6xEXLA3	Ibw6xChr08
Iba4xChr04	Iba4xEXLB1	Ibw6xEXLB2	Ibw6xChr04
Iba4xChr04	Iba4xEXLB1	Ibw6xEXLB7	Ibw6xChr10
Iba4xChr04	Iba4xEXLB3	Ibw6xEXLB1	Ibw6xChr04
Iba4xChr10	Iba4xEXLB4	Ibw6xEXLB4	Ibw6xChr10
Iba4xChr10	Iba4xEXLB5	Ibw6xEXLB6	Ibw6xChr10
Iba4xChr10	Iba4xEXLB6	Ibw6xEXLB1	Ibw6xChr04
Iba4xChr10	Iba4xEXLB6	Ibw6xEXLB7	Ibw6xChr10
Iba4xChr10	Iba4xEXLB7	Ibw6xEXLB8	Ibw6xChr10
Iba4xChr10	Iba4xEXLB7	Ibw6xEXLB11	Ibw6xChr10
Iba4xChr10	Iba4xEXLB8	Ibw6xEXLB12	Ibw6xChr10
Iba4xChr10	Iba4xEXLB8	Ibw6xEXLB9	Ibw6xChr10
Iba4xChr14	Iba4xEXLB9	Ibw6xEXLB13	Ibw6xChr14
Iba4xChr04	Iba4xEXLA1	Ibw6xEXLA1	Ibw6xChr04
IbaNHchr04	Iba6xEXLA2	Ibw6xEXLA2	Ibw6xChr04
IbaNHchr08	Iba6xEXLA3	Ibw6xEXLA3	Ibw6xChr08
IbaNHchr04	Iba6xEXLB1	Ibw6xEXLB7	Ibw6xChr10
IbaNHchr10	Iba6xEXLB4	Ibw6xEXLB9	Ibw6xChr10
IbaNHchr10	Iba6xEXLB5	Ibw6xEXLB1	Ibw6xChr04
IbaNHchr04	Iba6xEXLB1	Ibw6xEXLB1	Ibw6xChr04
IbaNHchr10	Iba6xEXLB5	Ibw6xEXLB7	Ibw6xChr10
IbaNHchr10	Iba6xEXLB6	Ibw6xEXLB11	Ibw6xChr10
IbaNHchr10	Iba6xEXLB6	Ibw6xEXLB4	Ibw6xChr10
IbaNHchr10	Iba6xEXLB6	Ibw6xEXLB8	Ibw6xChr10
IbaNHchr10	Iba6xEXLB7	Ibw6xEXLB12	Ibw6xChr10
IbaNHchr10	Iba6xEXLB7	Ibw6xEXLB9	Ibw6xChr10

IbaNHchr14	Iba6xEXLB8	Ibw6xEXLB13	Ibw6xChr14
IbaNHchr04	Iba6xEXLA1	Ibw6xEXLA1	Ibw6xChr04

Table S4. The protein sequences of different conserved motifs.

Motif	Length	Motif Consensus
motif1	41	DILAVEVYZKESYEWIAMRRVYGAVFDLQNPPKGELKVRFL
motif2	32	AGCGACYQVRCKNPALCSEEGTKVVVTDNGEG
motif3	23	LAIKIHDQSNYPGYLAILPLNQG
motif4	21	GACGYGDYGRTVNGGKVSAAS
motif5	21	ANDLFAKGVVDVEYRRVPCNY
motif6	21	SSKNIIPDDWKAGVTIDTGIQ
motif7	21	CYSQTYPSKATYYTTPDGMGT
motif8	15	TDFILSYRAYRKMAK
motif9	15	QGCTLLCIFLLLPAL
motif10	6	RLYKNG
motif11	33	RSTRGGAIWETSAAPSGPLQFRLVVTAGFDGKW
motif12	50	MQCFTWIRERFSGPNVQFCVIGDGWEECEAAEAMRWPFVKIDPCSSKYHR
motif13	15	ITDIAQEGCSPPDDD
motif14	50	IFPHSATVLQKESSQKCTEIYEGVHSLTIMDQKMNVFIWDMDETLILLKS
motif15	50	RHRVIAQKYKKGLHSILNGDMIKCWNDLYDETDSYTDRWLSSARTCLEQC
motif16	50	PIDANDGQGTKYQHVNVLVTSGTLIPSLVKCLLFRLGDIISYENVYSAWD
motif17	8	SSGQKWIE
motif18	6	MAFSLK
motif19	21	QTEIIAVDVAPVGFPNWSFMS
motif20	21	SAVAGGRAVPIGKLSSVQPSG

Table S5. The primer pairs of selected genes for qRT-PCR.

Gene Name	Primer Name	5'-3' sequence	Products
Iba6xEXLA3	F-LA3	CGAAGAAACAAGCCAGAAACCT	155bp
	R-LA3	CTCTGCTCGTGTCCCAAATAG	
Iba6xEXLB2	F-LB2	CTGCCAGCTGCGTTCTGCTA	146bp
	R-LB2	AGAGACGGCTTGAGGCAGCT	
Iba6xEXLB8	F-LB8	GGAGCTTGTGGGTTTGGTGAC	151bp
	R-LB8	TCCTCGTACCTTCCTCGTTGC	
